# Considering Spatial Scale and Reproductive Consequences of Habitat Selection when Managing Grasslands for a Threatened Species

**DOI:** 10.1371/journal.pone.0156330

**Published:** 2016-06-20

**Authors:** Scott F. Pearson, Shannon M. Knapp

**Affiliations:** 1 Washington Department of Fish and Wildlife, Wildlife Research Division, 1111 Washington St. SE., Olympia, Washington, 98501, United States of America; 2 Statistics Consulting Lab, Bio5 Institute, University of Arizona, 1657 E. Helen St., Tucson, Arizona, 85721, United States of America; University of Sydney, AUSTRALIA

## Abstract

Habitat selection that has fitness consequences has important implications for conservation activities. For example, habitat characteristics that influence nest success in birds can be manipulated to improve habitat quality with the goal of ultimately improving reproductive success. We examined habitat selection by the threatened streaked horned lark (*Eremophila alpestris strigata*) at both the breeding-site (territory) and nest-site scales. Larks were selective at both spatial scales but with contrasting selection. At the territory scale, male larks selected sparsely vegetated grasslands with relatively short vegetation. At the nest-site scale, female larks selected sites within territories with higher vegetation density and more perennial forbs. These nest-site scale choices had reproductive consequences, with greater nest success in areas with higher densities of perennial forbs. We experimentally manipulated lark habitat structure in an attempt to mimic the habitat conditions selected by larks by using late summer prescribed fires. After the burn, changes in vegetation structure were in the direction preferred by larks but habitat effects attenuated by the following year. Our results highlight the importance of evaluating habitat selection at spatial scales appropriate to the species of interest, especially when attempting to improve habitat quality for rare and declining species. They also highlight the importance of conducting restoration activities in a research context. For example, because the sparsely vegetated conditions created by fire attenuate, there may be value in examining more frequent burns or hotter fires as the next management and research action. We hope the design outlined in this study will serve as an integrated research and management example for conserving grassland birds generally.

## Introduction

When attempting to identify the mechanisms responsible for a species’ overall population decline, conservation biologists often use modeling approaches to first identify the relative importance of different vital rates like fecundity and survival to declines (e.g., [1–3] and then attempt to identify the mechanisms responsible for the depressed vital rate(s). With this approach, managers can employ management actions most likely to reverse negative population trends. For birds, adult and juvenile survival is often difficult to influence through management [4, 5] and consequently management activities tend to focus on annual fecundity [3].

Low fecundity is often the result of egg and nestling predation [6, 7]. To reduce predation rates, managers can use techniques that change predator behaviors [8], reduce their numbers [9, 10], or prevent them from accessing nests, chicks and/or adults [11, 12]. Alternatively, managers can manipulate habitat to create conditions that have lower predation rates. For some species there is an apparent relationship between habitat characteristics and nest success [13–15] and habitat conditions can have a positive effect on a species life-time reproductive success [16].

Habitat selection by migratory or semi-migratory territorial passerine birds is often viewed as a hierarchical process [17] where males arrive on the breeding grounds first and establish territories by active territorial defense and visual and auditory displays. Females arrive on the breeding grounds after males and select among males or territories for their social mate and select nest site locations. Because of this pattern, attempts to improve or increase suitable habitat for a rare of declining species should consider the various scales of habitat selection and the reproductive consequences of those choices [18].

The streaked horned lark (*Eremophila alpestris strigata*) is a partially migratory territorial subspecies associated with sparsely vegetated grassland habitats [19–22] and is listed as Threatened under the U.S. Endangered Species Act [23], and it is listed as endangered by Canada [24] by the state of Washington USA. The breeding range of streaked horned lark has contracted since the late 1800s, with local extirpation from the northern and southern extremes of its range [20–22, 25]. Streaked horned lark populations in Washington State USA are declining rapidly [3] and conservation efforts are needed that target both survival and fecundity [3]. Information is needed on habitat selection at various spatial scales to inform habitat management and restoration activities. Because low streaked horned lark fecundity is driven primarily by high rates of nest predation [12, 26], we would expect larks to select nest sites with characteristics that mitigate predation risks. For example, vegetation that helps conceal nests results in improved nest success for some species but not others [27, 28].

In an attempt to identify suitable habitat conditions that might influence streaked horned lark habitat use patterns and nest success, we examined habitat characteristics at territorial (10^3^ m^2^) and nest site (10^−1^ m^2^) scales within nine occupied sites over three years in Washington and northern Oregon. The study sites spanned the range of sites used by streaked horned larks in the northern half of its range including sandy island habitats on the lower Columbia River, coastal dune habitats on the Washington coast and native and airport grasslands in the Puget lowlands. These sites consist of grassland (10s to 100s of hectares in size) within a matrix of coniferous forests or adjacent to open water. We move beyond describing the distribution of the lark within occupied sites, by comparing habitat characteristics between high and low use portions of occupied sites. At the nest scale, we compare selected nest sites to nearby random sites (within the same territory) and we evaluate the reproductive consequences of this choice. Finally, we experimentally examine the effects of prescribed fire on lark abundance and habitat condition to help us assess the effectiveness of this management tool for improving lark habitat using the territorial habitat variables selected by larks as identified in the first part of this study. This study design allowed us to examine the following questions: (1) are streaked horned larks selective in habitat use at either the nest site or territory scales?, (2) do the nest site choices have reproductive implications?, and (3) does prescribed fire result in habitat conditions preferentially used by larks? Finally, this work serves as a case study of an approach for conducting research in a management and conservation context, especially for grassland birds.

## Methods

### Study species

The streaked horned lark is a partially migratory and genetically distinct subspecies [29–31]. In more northerly sites in Washington males arrive on the breeding grounds in late February and defend 'all-purpose territories' in which all breeding season activities occur, e.g., courtship, mating, nesting and foraging. Females arrive a two to three weeks after the males and select among territorial males and ultimately select nest sites. At the time of this study, the streaked horned lark was not listed as endangered or threatened by the Washington state or by the federal government.

### Study sites

The grasslands and oak woodlands of the Willamette Valley-Puget Trough-Georgia Basin ecoregion in British Columbia, Canada, and the states of Washington and Oregon are some of the most endangered ecosystems in the region [32]. The streaked horned lark was apparently closely tied to this ecosystem historically [33–36]. The current lark breeding range includes agricultural habitats and grasslands of the Willamette Valley of Oregon, dredge deposition islands along the lower Columbia River, southern Washington coastal dune habitats, and grasslands located on airfields or native prairies in the Puget lowlands near Olympia and Tacoma, Washington [22, 37]. Within this range, larks use relatively large open expanses of grass and forb dominated habitat or smaller patches of habitat adjacent to open water [37].

**Fig 1:**
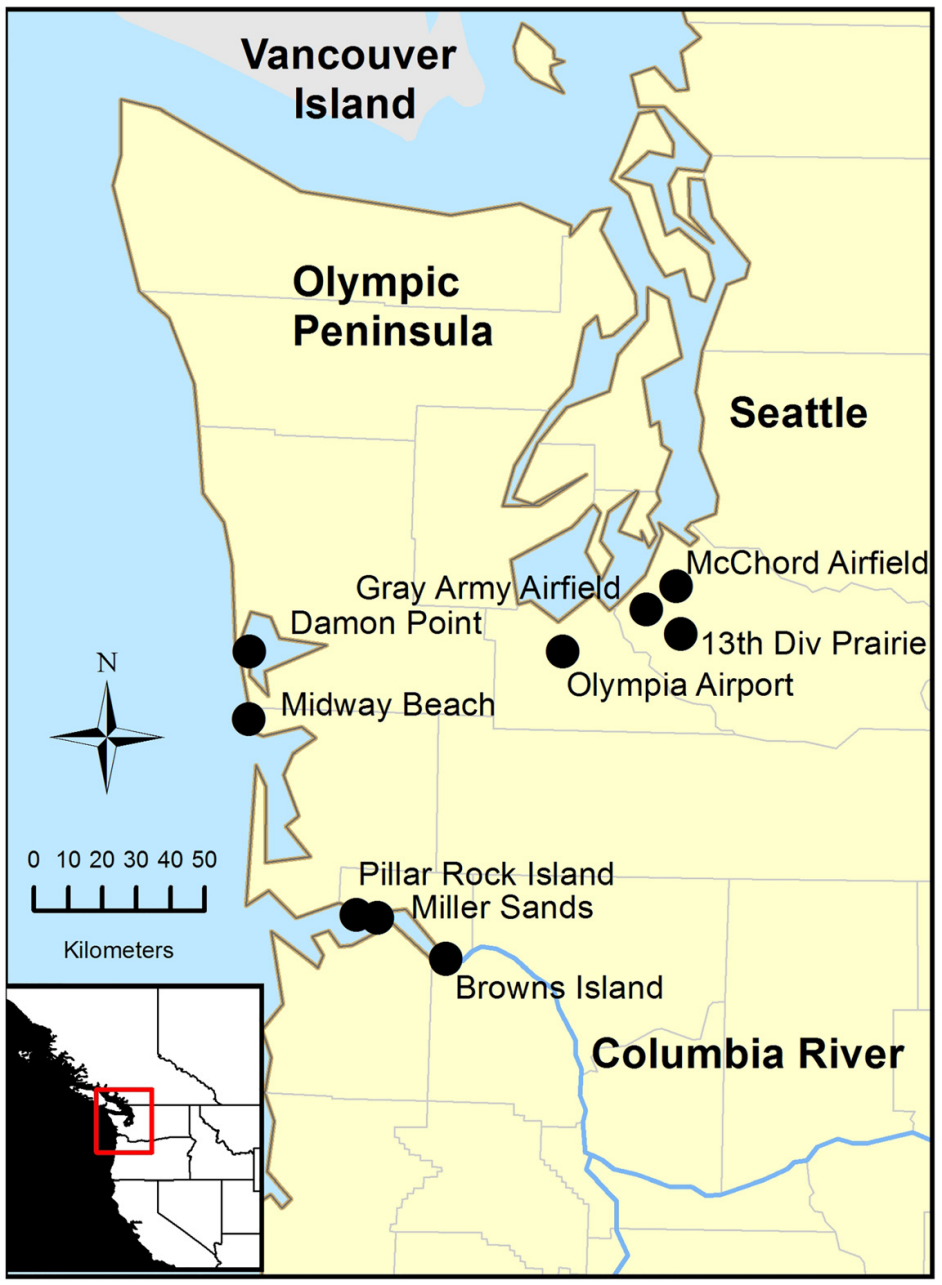
Study sites. Location of nine study sites in the Puget lowlands, Washington State coast, and lower Columbia River.

Locations of breeding larks were identified by searching suitable expanses of grass and forb dominated habitats within the historic range of the subspecies in Washington and on Columbia River islands of Oregon and Washington [20, 21]. Of the 17 occupied sites in Washington and along the lower Columbia River that were known at the time of the study, we selected 9 study sites along the Washington coast (n = 2), lower Columbia River (n = 3) or in the Puget lowlands (n = 4) that represent the diversity of habitat types used by the subspecies in the northern half of its range, had access, and, for the most part, had larger population sizes (Table 1, Fig 1). All of the four Puget lowland sites were located on glacial outwash soils that were historically dominated by Puget prairies [38, 39]. Three of these four Puget lowland sites were located on military or civilian airports and the third, 13^th^ Division Prairie, was located on a native prairie. All sites were dominated by grasses and forbs and the relative composition and proportion of native and non-native plants varied considerably within and among sites [39, 40]. The Columbia River island sites consisted of accreted sandy soils and sites created by the deposition of dredge materials deposited on the shore and on islands when deepening and maintaining the Columbia River navigation channel. The extent of grass and forb habitat varied depending on time since deposition. Joint Base Lewis-McChord, Port of Olympia, Port of Shelton, Washington Departments of Natural Resources and State Parks, and Oregon Division of State Lands granted access to our study sites and allowed us to conduct our fire experiment (see below).

**Table 1:** Locations of study sites, years monitored and approximate number of lark territories per site in Oregon and Washington, USA.

Site	Habitat type	Latitude/ Longitude	Approx. number of Territories	Years
**Puget Lowlands**				
13^th^ Division Prairie	Prairie	47° 01' N 122° 26'W	10–18	2002–2005
Gray Army Airfield^1^	Airfield	47° 05' N 122° 34'W	30–31	2002–2005
McChord Air Force Base^2^	Airfield	47° 08' N 122° 28'W	28–31	2002, 2004, 2005
Olympia Airport	Airfield	46° 58' N, 122° 53'W	18–25	2002
**Washington Coast**				
Damon Point	Coast	46° 56'N, 124° 06' W	12–17	2004, 2005
Midway Beach	Coast	46° 46'N, 124° 05' W	12–21	2004, 2005
**Columbia River (Oregon and Washington)**				
Browns Island	Island	46° 08'N, 123° 18' W	8–13	2004, 2005
Miller Sands	Island	46° 14'N, 123° 38' W	3–6	2005
Pillar Rock Island	Island	46° 15'N, 123° 35' W	6–10	2005

^1^Only the north and south ends were used in 2002 and 2005 and the entire airfield was used in 2003 and 2004.

^2^Results are only from the intensive study area in the northeast portion of the airfield.

### Identifying low and high use areas within territories

The number of territories monitored per site ranged from 4–26 (Total approximately 149; Table 1) depending on the size of the site or our ability to access the entire site (McChord AFB had restricted access). Within the intensive study areas, locations of adult male and female larks were mapped by walking transects spaced 150 m apart with the goal of surveying the entire study site or the entire portion of the site where access was permitted. While walking transects, we plotted the locations of birds on orthographic photos (46 cm resolution) carried in the field. We used visible landmarks to accurately place each bird within approximately 10 m of its actual location. These transects were visited approximately six times per year by trained biologists between mid-April and 15 July and between sunrise and 11:00 am Pacific Standard Time in 2002–2005. The lark location data were digitized in ArcGIS. To compare habitat characteristics associated with high and low use areas, we applied fixed kernel density estimates to lark localities using the kernel estimator in ArcGIS 9.0. We randomly located one habitat sampling point in both the high and the low density areas (upper and lower quantiles of kernelled density) of each territory. High density areas closely approximated the core of lark territories (Table 1 provides a count of the number of territories per site). All vegetation measuring points were > 75 m apart.

### Habitat measurements, territory scale

To measure vegetation characteristics associated with high and low density areas within a site, we positioned two 25 m perpendicular meter tapes that crossed at their midpoints over the random sampling points. The tapes were oriented north-south and east-west. We used the point intercept method [41]. At each meter mark we leveled our point intercept device using a bubble level (to reduce potential visual bias of placement and maintain uniform perpendicular angle—all sampling plots were relatively flat) and the pin was dropped. A single drop that resulted in more than one “hit” by the pin for a given plant species was recorded as a single hit for that species per pin drop or 100% cover. Plant species were put into the functional groupings (territory and nest vegetation variables) in Table 2. For all analyses, total hits for a given functional group and for ground cover (Table 2) within a plot were divided by the total pin drops per plot (n = 52) to give a percent cover. We recorded all pin drops that did not touch a living plant as “unvegetated” drops. We also recorded the “total vegetation hits” which is the total number of grasses and forbs hit by the pin during all 52 drops within a plot. This variable includes all vegetative hits even if there were multiple hits by a given species during a single pin drop. At the ground level, we recorded whether the pin hit bare ground (rock, sand, dirt, shell), Moss/lichen/algae, or thatch (dead vegetation). Finally, we recorded the height of the tallest plant touching the pin for each drop and used the average height per plot for all analyses. Only drops that hit a plant were included in the estimate of vegetation height.

**Table 2:** Habitat variables measured at nest (1 m^2^) and or territorial plot scales (25 m^2^).

**Territory and nest vegetation variables (functional groups)**				
**Variable**	**Annual perennial**	**Native/non-native**	**Form**	**Unit**
Grasses	Annual	Native		Percent
		Non-native		Percent
	Perennial	Native	Caespitose or tuft	Percent
		Non-native	Rhizomatous	Percent
		Non-native	Tuft	Percent
Forbs	Annual	Native		Percent
		Non-native		Percent
	Perennial	Native		Percent
		Non-native		Percent
**Other variables**				
**Variable**	**Description**			**Unit**
Vegetation density	Average total vegetative hits per pin drop per plot			Average count
Unvegetated	Pin drops that don’t touch live vegetation			Percent
Ground cover	Bare ground (rock, sand, dirt, shell)			Percent
Ground cover	Moss/lichen/algae			Percent
Ground cover	Thatch			
Vegetation height	Height of tallest plant touching or adjacent to pin drop			cm
**Nest site only measurements**				
Cover from above	concealment of the nest by foliage when looking down on it from a distance of 1 m			Percent
Base plant (7 categories)	Annual forb, perennial forb, annual grass, rhizomatous grasses, other perennial grasses, native rush, and shrub			Categorical

### Locating nests

Within territories, we searched for nests from early April to mid-August in 2002–2005 (Table 1). Nests were located by observing adults, flushing incubating or brooding adults, and searching appropriate habitat. Nests were found during the nest building, incubation, and nestling stages. Nest stage (nest building, pre-laying but post-building, incubation, and nestling) was recorded every 1–3 days, and every day or every other day near expected hatch and fledging dates. Expected hatch and fledging dates were estimated using known hatch or egg-laying dates and the following intervals: 1 egg laid per day (thus, the number of eggs in a clutch equals the length of the laying period), an incubation period of 12 days, and nestling period of 9 days [19]. For nests found during the nestling period or with unknown hatching dates, hatching and fledging dates were estimated using photographic reference images of known aged chicks that depicted daily changes in down and contour and flight feathering and because feathering changes so rapidly over the 9-day nestling period we could estimate chick age ± 1 day. Nests were considered successful if adults were observed with fledglings in their territory (territories were mapped) within 3–4 days of fledging (chicks are unable to fly during this period) or nests were found empty with signs of fledging (flattening of nest cup and fecal droppings in or near nests) on or after the expected fledging date. Nests with signs of predation were counted as unsuccessful. Signs of predation included damaged eggs, blood or feathers in or near a nest, and nests found empty during incubation or during the nestling period when nestlings were too young to have fledged (< 8 days post-hatching). Nests were considered abandoned if a nest and its contents were intact and no adults were observed after 2–3 visits and > 7 days had passed after the estimated fledge date. Nests that were abandoned and later predated were considered abandoned.

### Habitat measurements, nest site scale

For nest site habitat measurements, we again used the point intercept method and used a 1 m wooden pin frame where a pin was dropped every decimeter (starting at zero) through holes drilled in the frame following Barbour et al. [42]. For each pin drop, we recorded the same variables in the same functional groups as described above for territories. For each nest, the frame was centered on the nest so that the axis was oriented north-south and 11 pin drops were conducted and then the frame was oriented east-west and 11 pin drops were conducted. Nonuse nest sites were located using a random distance (> 1.5 m but ≤ 10 m from the nest) for the center of the plot and random azimuth from a given nest. These “nonuse” nest sites were located within the same vegetation type (grassland) and were located within the same male's territory as the nest site. The assumption is that the random site could have been selected by the female but was not. We used the same sampling protocol for nonuse nest sites as was used for nest sites. Females usually build their nests on the north side of a plant [43] and we recorded the functional group of the “base plants” to examine their potential influence on nest success. Finally, we estimated the percent of the nest concealed by vegetation when looking at the nest from 1 m above. The concealment and base plant variables were only measured at nests and not at random plots.

### Fire experiment

On the native 13^th^ Division Prairie, we used a Before-After, Control-Impact (BACI) design to examine the effects of fire on lark habitat and abundance. We randomly established 12 treatment and control plots (50 x 50 m squares) in areas with moderate to low lark density as determined by fixed kernel density estimates. Plots were burned on 8 September 2004. Vegetation was measured before (late June–early July 2004), immediately after (late September–early October 2004) the burn, and during the breeding season after the burn (late June–early July 2005). Bird plots were surveyed 10 times prior to the burn (3 August– 3 September 2004), five times immediately after the burn (9 September– 1 October 2004), and fifteen times during the breeding season following the burn (6 April– 30 July 2005). Vegetation characteristics of burn and control plots were measured using the methods described for territories above with the 25 m point intercept transects located in the center of each burn and control plot and again oriented north-south and east-west. Bird abundance was measured by walking a transect through the center of each plot. The starting end of each transect was alternated with each visit and the order in which the plots were visited was also alternated. Upon arrival at a treatment or control plot, observers would spend 5 minutes on the edge of the plot facing the plot center, observers would then walk to the center of the plot and spend 5 minutes in the center slowly scanning the entire plot, and then continue across the plot to the far side where she/he would spend 5 minutes on the outside edge looking back into the plot.

### Data analyses

We used MANOVA to test whether there was a difference in the vegetation characteristics (Table 2) between high-use and low-use areas of a territory. Because vegetation sampling was done across a range of sites and years, we treated combinations of Site and Year as a block effect. After eliminating the data for 13th Division Prairie in 2003 (there were only 3 observations each for the high-use and low-use areas but higher sample sizes for this site in 2002), there were a total of 10 site-year blocks. We then screened out variables based on distribution or relationship to other variables. Three variables (native perennial caespitose grass, non-native—tuft forming perennial grass, and shrubs/trees) were eliminated because of their very limited range and/or highly skewed distribution. For example, 184 of the 241 observed non-native, tuft forming perennial grass values were zero and 90% of the observations were less than 0.10, but values as large as 0.77 were observed. Also, because thatch, bare ground (includes rock and shell), and moss/lichen/algae must sum to 1, any one of these variables is a linear combination of the other two, so we chose to use only bare ground and moss/lichen/algae in our analysis. The logic for removing thatch was because this variable was likely related to total vegetation density and the density of grasses in particular, which are both already included in the analysis. The analysis was conducted using SAS PROC GLM (SAS 9.4, SAS Institute, Cary NC)).

We used MANOVA to test whether there was a difference in the vegetation characteristics between nest sites and nearby random points. We used the same set of vegetation variables in this analysis as we used for the comparison between high-use and low-use areas of territories. Because random points were paired with nest points we blocked by the nest-random pair (this is analytically equivalent to conducting a paired Hotelling T^2^ test or conducting a one-sample Hotelling T^2^ test on the differences in vegetation measurements between a nest site and the paired random site).

To examine the effects of habitat variables on nest survival, we used the logistic exposure method [44–47]. We ran analyses for non-linear mixed models using SAS PROC NLMIXED (SAS 9.4, SAS Institute, Cary NC). The SAS code used follows that in Appendix 4 in Rotella et al. [46]. We used the effective sample size (“n_ess_”;[46]) when computing AIC_c_. n_ess_ equals the sum, over all nests, of the number of days each nest was under observation and survived, plus the number of observed failures). AIC_c_ is a small-sample bias adjustment (corrected) AIC [48].

Before testing for habitat effects on nest survival, we first establish a baseline model. We considered the following variables for the baseline model: Site (as both a fixed and a random effect), Date (with 0 = April 1; both linear and quadratic), Nest Age (both linear and quadratic), Year (both as a fixed and a random effect), and a categorical Habitat Type with either 4 levels (prairie, airfield, coastal dune, and island) or 2 levels (combined prairie-airfield and combined coast-island) (S1 Table). These habitat types are essentially ecological strata that differ spatially (island = Columbia River, coast = Pacific coast, and prairie and airport = Puget lowlands), in vegetation composition, and in management activities (e.g., active mowing and restoration on airports and prairies). In order to compare models using AIC, all models must be run on the same subset of data. Unfortunately, we didn’t have Nest Age for all nests. Consequently, for the baseline analysis we used only nests with Nest Age and from Sites 13^th^ Division Prairie, Damon Point, Gray Army Airfield, McChord Airfield, Midway Beach, Olympia Airfield, Browns Island (Pillar and Miller islands had only a single nest each). We first examined each of the single-variable models as well as a constant (intercept-only) model.

**Fig 2:**
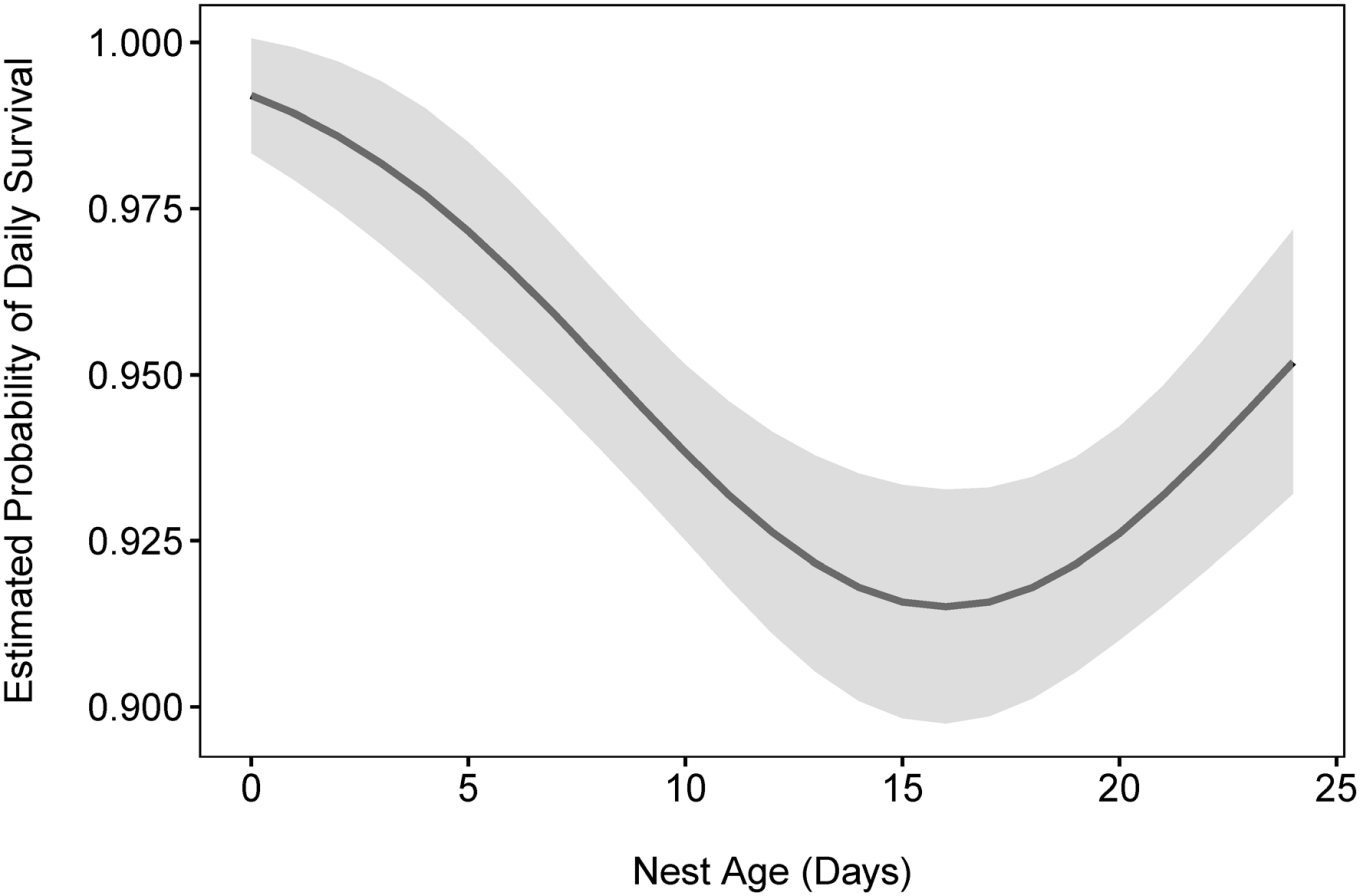
Nest age and survival. Predicted daily streaked horned lark nest survival (days) using the equation from the baseline model. Nests included in the model were from sites located in the Puget lowlands, the lower Columbia River and the Washington coast (n_ess_ = 2269). Daily nest survival decreases shortly after hatching and then increases again closer to fledging (egg laying = 2–5 days depending on clutch size—mode = 3, incubation = 12, nestling = 7–9).

Quadratic Age ranked highest followed by models with habitat (2 levels and 4 levels) and a fixed-effect year. At the next stage we added each of these effects to the model with quadratic age. The model with quadratic age was still selected as best, followed by Quadratic Age + 2-level Habitat (ΔAIC_c_ = 1.238), Quadratic Age + 4-level Habitat (ΔAIC_c_ = 1.489), and Quadratic Age + fixed-effect Year (ΔAIC_c_ = 4.372) (S1 Table). Thus, in the interest of parsimony, we opted to use the model with only Quadratic Age as our baseline model (Fig 2).

Once we had established a baseline model, we added vegetation variables (Table 2) to the baseline looking for improved fit (using AIC_c_ as criteria). As only models built on the same subset of data should be compared using AIC_c_, and because there were a large number of vegetation variables each with missing values, to subset the data to only those observations with all vegetation variables could have dramatically reduced sample size, we chose to first test each model set against the baseline (with the proper subset of data), then compare any models that showed an improvement over the baseline at the necessary data subset. The full set of models examined is listed in Table 3. The models selected were designed to examine nest concealment from potential visual predators, habitat structure, the influence of the base plant (again, nearly all nests are placed on the north side of a plant), and the relative influence of native and non-native annual and perennial forbs and grasses on nest success. We examined the influence of native prairie structure on nest success. Native prairies were dominated by native bunchgrasses (caespetose or tuft growth form vs. rhizomatous turf forming grasses) and forbs and by a more classic structure (sparsely vegetated with relatively short vegetation). The hypothesis going into this study was that larks would select sites that were sparsely vegetated and where it was easy to walk between plants (plants in a tuft or caespetose growth form vs. dense sod forming rhizomatous grasses), which is their primary mode of moving while on the ground [19].

**Table 3:** Sets of models tested to examine the effects of concealment, vegetation structure, baseplant, and native and non-native plants on lark nest success. *k* denotes the number of parameters in the model. Δ*AIC*_*c*_ is given relative to the respective baseline model; thus negative values of Δ*AIC*_*c*_ indicate the given model is preferred over the baseline model.

Variables added to baseline model	*k*	Δ*AIC*_*c*_
**1—Concealment (*n***_***ess***_ **= 2121)**		
Cover above	4	1.716
Cover above + Cover above^2^	5	1.145
**2—Vegetation Structure (1) (*n***_***ess***_ **= 2149)**		
Perennial forb + Native perennial caespitose grass + Non-native perennial grass tuft	6	4.486
Perennial forb + Native perennial caespitose grass + Non-native perennial grass tuft + Perennial forb^2^ + Native perennial caespitose grass^2^ + Non-native perennial grass tuft^2^	9	8.100
**3—Concealment and Vegetation Structure (1) (*n***_***ess***_ **= 2121)**		
Cover above + Perennial forb + Native perennial caespitose grass + Non-native perennial grass tuft	7	6.584
**4 –Base plant (*n***_***ess***_ **= 2029)**		
Base plant (as 7 categories in Table 1, shrub = reference category)	9	9.559
Base plant = perennial forb (native + non-native)	4	1.167
Base plant = perennial grass (all functional groups)	4	1.624
**5—Vegetation Structure (2) (*n***_***ess***_ **= 2029)**		
Vegetation density	4	1.738
Vegetation density + (Vegetation density)^2^	5	3.506
Non-vegetated hits	4	1.457
Non-vegetated hits + (Non-vegetated hits)^2^	5	3.450
Average vegetation height	4	1.467
Average vegetation height + (Average vegetation height)^2^	5	3.474
Bare ground	4	2.000
Bare ground + Bare ground^2^	5	3.986
(Moss/lichen/algae + thatch)	4	2.000
(Moss/lichen/algae + thatch) + (Moss/lichen/algae + thatch)^2^	5	3.986
**6—Native & Non-Native Perennial Forbs (*n***_***ess***_ **= 2149)**		
Native perennial forb	4	0.773
Native perennial forb + Native perennial forb^2^	5	2.111
Non-native perennial forb	4	-3.754
Non-native perennial forb + Non-native perennial forb^2^	5	-1.752
Native perennial forb + Non-native perennial forb	5	-2.375
Native perennial forbs + non-native perennial forbs + Native perennial forbs × Non-native perennial forbs	6	-0.895
Perennial forbs (native plus non-native)	4	0.988
Perennial forbs + Perennial forbs^2^	5	1.267
**7—Native & Non-Native Perennial Grasses (*n***_***ess***_ **= 2149)**		
Native perennial caespitose grass	4	1.668
Native perennial caespitose grass + Native perennial caespitose grass^2^	5	2.837
Non-native perennial rhizomatous grass	4	1.984
Non-native perennial rhizomatous grass + Non-native perennial rhizomatous grass^2^	5	3.984
Native perennial caespitose grass + Non-native perennial rhizomatous grass + Native perennial caespitose grass × Non-native perennial rhizomatous grass	6	5.060
**8—Native & Non-Native Annual Forbs (*n***_***ess***_ **= 2149)**		
Native annual forbs	4	0.755
Native annual forbs + Native annual forbs^2^	5	2.754
Non-native annual forbs	4	1.560
Non-native annual forbs + Non-native annual forbs^2^	5	3.024
Native annual forbs + Non-native annual forbs	5	2.348
Native annual forbs + Non-native annual forbs + Native annual forbs × Non-native annual forbs	6	4.365
**9—Native & Non-Native Annual Grasses (*n***_***ess***_ **= 2149)**		
Native annual grasses	4	1.945
Native annual grasses + Native annual grasses^2^	5	3.693
Non-native annual grasses	4	2.006
Non-native annual grasses + Non-native annual grasses^2^	5	4.001
Native annual grasses + Non-native annual grasses	5	3.946
Native annual grasses + Non-native annual grasses + Native annual grasses × Non-native annual grasses	6	5.958

To examine if prescribed fire resulted in habitat conditions preferred by larks (as determined by the analysis of vegetation characteristics between High-Use and Low-Use area of territories), we ran a 2-factor ANOVA with Time (3 levels: 0 = Pre-fire, 1 = immediately after fire, 2 = breeding season after the fire, Treatment (2 levels: Burn, Control) and a Time-Treatment interaction. We also estimated the contrasts for the difference between the change in the response for the Burned Treatment and the change in the response for the Control; this was done both for the immediate response to the burn and for the breeding season following the burn because we were really interested in the breeding season effects. If the Time-Treatment interaction was statistically significant (α = 0.05), we then used a *t*-test to examined whether the contrast was significantly different from 0; that is, whether the change in the response for the Burned Treatment was significantly different than the change in the response for the Control. For these analyses we used the following set of vegetation variables as the response: moss/lichen/algae, bare ground, annual forbs, perennial forbs, annual grasses, perennial rhizomatous grasses, non-vegetated drops, total vegetation hits. We selected these variables because they were thought to be important to larks at the territorial scale, to be consistent with our high-low use analysis, and to exclude variables with limited data or skewed distributions.

Despite the sparse data [a total of 4 birds detected over 120 surveys on 12 plots Pre-burn and 18 birds detected over 191 surveys on those 12 plots (Summer following the burn)], we tested to see whether there was a Treatment-Time interaction effect in lark abundance. We used the pcount function in the R package 'unmarked' [49] to fit the N-mixture model of Royle [50] using time (Pre vs. Post2), treatment (control vs. burn), and a treatment-time interaction for abundance and a constant detection function.

## Results

### High use and low use areas at territory scale

The MANOVA results indicated a statistically significant difference in the multivariate vegetation characteristics between high-use and low-use areas at a territory scale (Wilk's Lambda = 0.47, *F*_9,222_ = 27.68, *p* < 0.0001). On average, high use areas had lower moss/lichen/algae, native perennial forbs, non-native perennial forbs, non-native perennial rhizomatous grasses, and lower average vegetation height than low use areas (Table 4). High use areas had higher percent cover of bare ground, annual grasses and non-vegetated hits (Table 4). There was virtually no difference between high and low-use areas in average cover of annual forbs (Table 4).

**Table 4:** Mean difference in vegetation height (cm) and percent cover of other habitat variables between nest and nearby random plots and between high and low lark density areas within breeding sites. P value is from the univariate test. Nest scale n = 287–288, Territory-site scale n = 118 low and 123 high density. Note that the nest site differences used a paired design and the high and low density territory differences were not paired.

Variable	Mean DifferenceNest—Random (SE)	Mean Difference High—low lark density (SE)	Nest—random P Value	High—low density P Value
Moss/lichen/algae	-0.06(0.01)	-0.06(0.03)	<0.0001	0.0277
Bare ground	-0.01(0.02)	0.25(0.03)	0.8683	<0.0001
Annual Forbs	0.01(0.01)	0.01(0.02)	0.6069	0.6551
Native perennial forbs	0.04(0.01)	-0.05(0.02)	<0.0001	0.0123
Non-native perennial forbs	0.01(0.01)	-0.11(0.02)	0.1734	<0.0001
Annual grasses	-0.03(0.02)	0.15(0.03)	0.1131	<0.0001
Non-native perennial rhizomatous grasses	0.01(0.02)	-0.27(0.03)	0.6018	<0.0001
Un-vegetated	-0.89(0.18)	9.72(1.09)	<0.0001	<0.0001
Vegetation height	6.59(3.24)	-11.33(1.49)	0.0543	<0.0001

### Nests & nearby random points

The MANOVA results indicated a statistically significant difference in the multi-variate vegetation characteristics between nest sites and nearby random points (Wilk's Lambda = 0.7736, *F*_9,277_ = 9.01, *p*-value < 0.0001). Univariate analyses indicated only 3 of the 9 vegetation variables differed significantly (α = 0.05) between nest sites and random sites: moss/lichen/algae and non-vegetated hits were lower on average at nest sites than at nearby random points, and native perennial forbs which was higher on average at nest sites compared to nearby random sites (Table 4). Average vegetation height was also higher, on average, at nest sites than at nearby random sites (mean difference: 6.59 cm ± 3.2 SE, *p*-value = 0.054, Table 4).

### Influence of habitat characteristics on nest success

**Fig 3:**
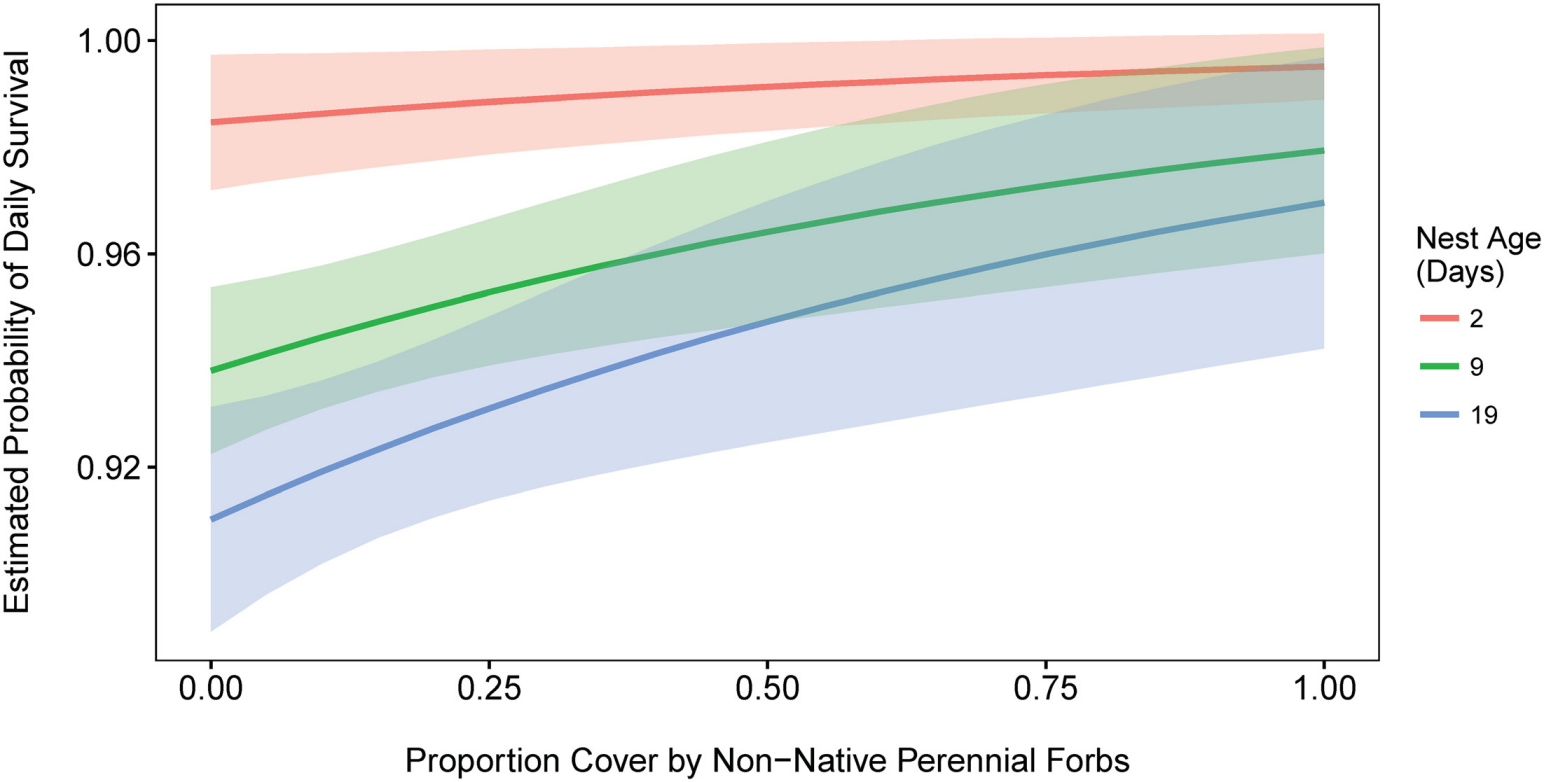
Influence of forbs on nest survival. Predicted relationship between percent cover of non-native perennial forbs and daily survival rate for 2, 9, and 19 day-old nests (n_ess_ = 2149). Model = baseline model + percent cover non-native forbs. Note that an increase in daily survival rate from 0.90 to 0.95 equals a 20% increase in overall nest survival.

We found evidence of perennial forb influence on nest success (see Table 3, Fig 3); in particular there was some evidence for an increase in nest success with increasing cover of non-native perennial forbs.

### Fire experiment

Estimates of the difference in the mean change for each vegetation variable in the burn treatment and the mean change in that vegetation variable in the control (contrasts) along with standard errors are given in Table 5. Of the vegetation characteristics examined, only perennial forbs, annual grass, non-vegetated hits, and average vegetation height showed a statistically significant Time-Treatment interaction effect (*p*-value < 0.05, Table 5), although the interaction effect for bare ground was marginal (*p*-value = 0.0516; Table 5). There was a statistically significant difference in the change of the vegetation characteristic between burned and control plots immediately post burn. However, there was no statistically significant difference in the change in these vegetation characteristics by the next breeding season (Table 5).

**Table 5:** Analysis results from our (BACI) experiment examining the short and longer-term effects of prescribed fire on habitat variables. The SE is the same for both estimates (Pre vs. immediately after the fire and Pre vs. the breeding season following the fire).

		Immediately post burnvs. pre-burn		Breeding season following burn vs. pre-burn		
Variable	Time × Treatment *p*-value	BACI effect estimate	*p*-value	BACI effect estimate	*p*-value	BACI effect SE
Moss	0.6024	0.0673	0.5981	0.1282	0.3182	0.1263
Bare ground	0.0516	0.1442	0.1283	0.2340	0.0166	0.0922
Annual forbs	0.8920	0.0288	0.7773	-0.0192	0.8503	0.1011
Perennial forbs	0.0002	-0.5256	< 0.0001	-0.1923	0.0956	0.1118
Annual grasses	< 0.0001	-0.7340	< 0.0001	0.0609	0.6394	0.1287
Perennial rhizomatous grasses	0.3459	-0.2500	0.1488	-0.1154	0.4922	0.1687
Unvegetated	< 0.0001	6.5000	< 0.0001	0.5000	0.6864	1.2263
Vegetation density	0.0012	-70.5000	0.0003	-28.8333	0.1043	17.2137

On burned plots, perennial forbs, annual grass, and vegetation density decreased initially, while each of these variables showed a mean increase on Control plots. In contrast, mean number of non-vegetated hits initially increased on the burn plots and decreased on the control plots. Thus the early response to the burn was in the direction preferred by larks (as suggested from the High-Low analysis) for perennial forbs, non-vegetated hits, and total vegetation height but not for annual grass; however, these effects had attenuated by the next breeding season.

**Fig 4:**
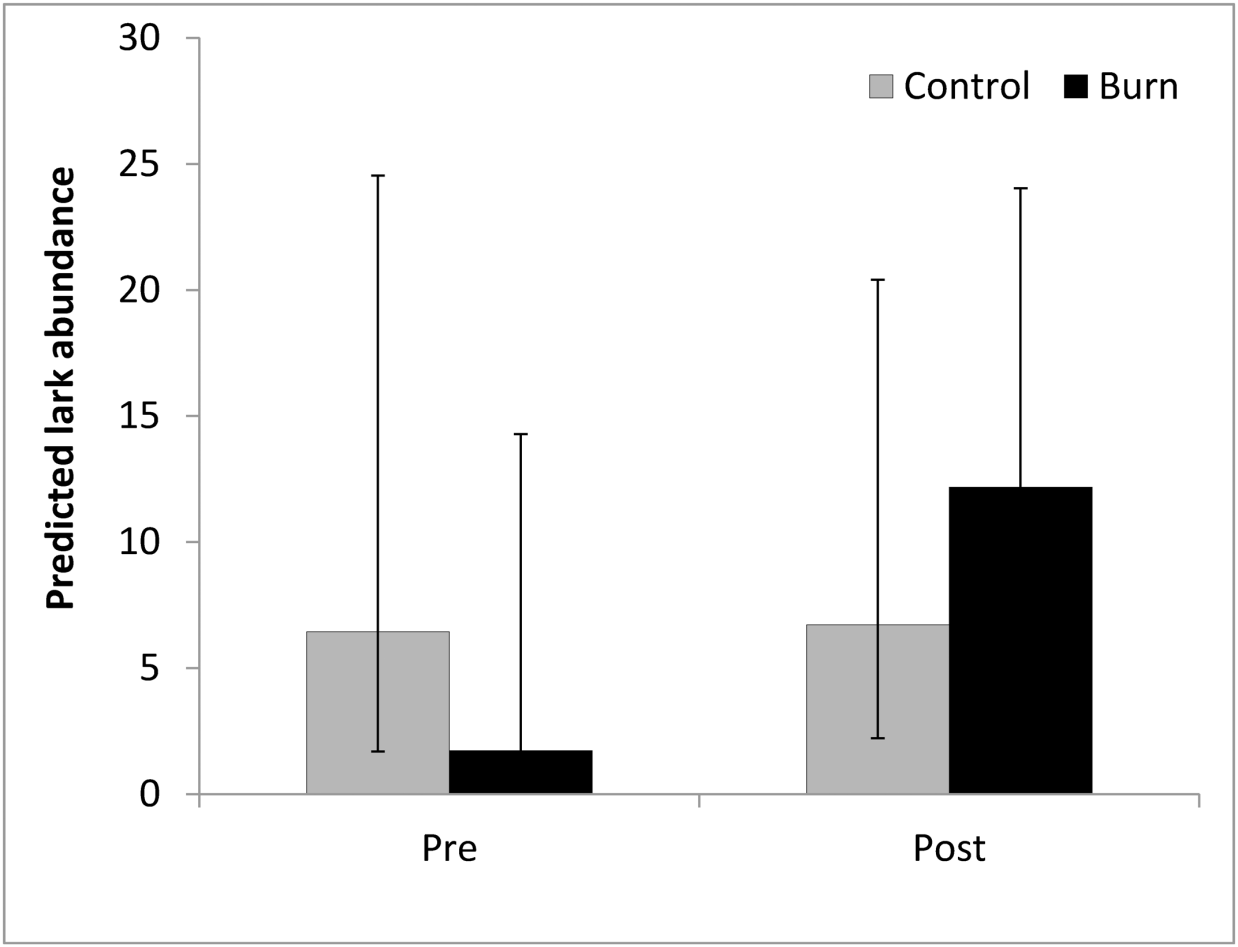
Influence of fire on lark abundance. Predicted lark abundance on treatments and controls before and in the breeding season following the prescribed fire treatment. Error Bars represent the 95% CIs.

The burn plots had a lower predicted abundance than the control plots pre-burn and a higher predicted abundance post-burn (Fig 4); however, the Treatment-Time interaction effect on abundance was not statistically significant (*p*-value = 0.1212) nor were the main effects of time (*p*-value = 0.9511) or treatment (*p*-value = 0.2435).

Although not the focus of our study, we also qualitatively examined the immediate post-fire response of larks to the fire treatment and found that they were not detected on control plots while five birds were detected on burn treatment plots.

## Discussion

Understanding the habitat characteristics selected by rare and declining species is critical to effective habitat management. Across its range, the horned lark uses grasslands composed of relatively short and sparse vegetation [51, 52]. Similarly, we found that streaked horned larks disproportionally used areas of a breeding site with more bare ground and with less and shorter vegetation. The importance of open sparsely vegetated grasslands may be the result of a variety of factors including a preference for open landscapes for flight and vocal displays, habitat specialization, and/or because Horned Larks walk through the grass rather than hopping [19], it may be easier to walk through environments with larger interstitial spaces between plants. However, at the nest site scale, females are selecting areas with relatively taller and denser vegetation and more perennial forbs than are typically used by larks at the larger site or territory scale. This suggests a very different micro-habitat selection process that might be driven by predation pressure. Collectively, these results indicate that larks are selective in their habitat choices at both the nest site and territorial scales but that there are potentially important differences between these choices at these different spatial scales.

Daily nest survival was higher during incubation, decreased fairly dramatically shortly after egg hatching, and then increased somewhat during the late nestling stage (Fig 2). It has been argued that predators use parental activity to locate nests [53] which may explain this increase in nest predation shortly after hatching when adult activity around the nest increase as adults begin to feed their young. However, changes in predation rates between nesting stages likely have both environmental (nest site) and behavioral (parental activity) influences [54, 55]. Female larks selected nest sites with higher vegetation density than nearby locations. These patches may help conceal the nest, yet we found no effect of concealment on nest success as some other researchers have found [28]. When accounting for differences in daily nest survival among nesting stages (our baseline model), we found an influence of an environmental factor, non-native perennial forbs. However, because we tested many models, it is possible that this relationship is a random chance (Type I error) and therefore recommend moving forward with management actions in a research context to see if this relationship holds.

Why might perennial forb cover positively influence nest survival if nest concealment and vegetation density did not? A predator’s effectiveness in locating nests may be influenced by a variety of vegetation characteristics including spatial heterogeneity, density, and species composition and interspersion. The architecture of perennial forbs may provide an additional element of camouflage to adult birds returning to the nest that is not awarded by vegetation density alone. Some perennial plants, like lupines (*Lupinus* spp.), appear to create high-contrast patterns. These patterns are not only created by their palmate leaves but also by the shadows that their leaves cast on the surrounding environment. These patterns may break up the bird’s outline thereby making it harder to track an already cryptic moving object. Alternatively, perennial forbs may increase habitat complexity of a grass dominated environment. This complexity may reduce predator search efficiency by impeding travel or inhibiting transmission of visual cues [56]. These hypotheses are worthy of additional study.

Our results have important management implications. If we are going to rely on habitat studies to inform management prescriptions, studies should consider different spatial scales of habitat selection and the influence of selection on vital rates. Our results suggest the need to manage for large grass and forb dominated landscapes that are sparsely vegetated and preferentially used by this subspecies. At the same time, our results indicate the value of maintaining or creating smaller forb-rich patches within these landscapes for nest sites that may help reduce predation rates and ultimately increase fecundity (Fig 3).

Ecosystem processes and disturbance regimes can be critical to creating and maintaining suitable habitat conditions for rare and threatened species [57, 58] (Schultz and Crone 2002, Warren and Büttner 2008). The ecosystem disturbance processes that maintained early successional habitat conditions used by the streaked horned lark in this region likely included flooding, fire, and sand accretion caused by flooding and long-shore currents depositing coastal sands. Larks readily move into the relatively barren landscapes created by the evaporation of ephemeral wetlands and into areas where coastal accretion is active [22]. However, many ephemeral wetlands in the region have been lost to draining, ditching and development. For example, approximately 57% of the Willamette Valley’s original wetlands have been lost [59]. Winter rain floods and spring snowmelt floods along the lower Columbia River, which prior to damming the main river and its tributaries could raise portions of the lower river by 20 to 30 feet [60, 61], and may have created early successional habitat used by streaked horned larks in the past through a process of scouring and deposition. These dams have also influenced patterns of accretion and erosion along Washington’s coast. Before dams, Columbia River floods carried sand to the delta where it was deposited in shoals. It was then picked up and moved northward to beaches by longshore currents. Prior to building the dams, the Columbia River carried about 12 million cubic yards of sediment to the Washington coast per year but this flow has been reduced by two-thirds since the building of the dams [62].

Grassland ecosystems throughout the world, including those in this ecoregion [63], were also historically maintained by natural and anthropogenic fire. Lightning, the other obvious cause for fires, occurs very infrequently in this area [64]. Native Americans used late summer and early fall fires (when we conducted our experimental burns) with a frequency of one to several years to prevent forest succession and to maintain grassland vegetation [63, 65, 66]. In the more recent past, human influences have reduced grassland fire frequency resulting in additional encroachment by trees and shrubs [67–69].

Because the processes that likely maintained lark habitat historically have been changed, it may be necessary to actively manage habitat for this species. Prescribed fire is one management tool that might result in the mosaic of habitat conditions needed by larks at both the nest site and territorial scales. Fire is currently being used to benefit various species associated with these unique grasslands including native plants and butterflies [70]. Given the success of prescribed fire in benefitting grassland birds generally, its use in this region may also prove beneficial. For example, prescribed fires in prairie fragments in the midwestern U.S. has created a mosaic of successional habitats that increased relative abundance of eastern meadowlark (*Sturnella magna*), grasshopper sparrow (*Ammodramus savannarum*) and western meadowlark (*Sturnella neglecta*) [71–73].

Using an experimental approach, we found that prescribed fire initially resulted in the sparsely vegetated landscape preferred by horned larks but most of those vegetation effects attenuated by the following nesting season. Other prescribed fire studies in the region also suggest a moderate reduction in total percent cover of vegetation in the growing season following a single burn [74], while another study suggests an increase in bare ground (particularly in the year following the burn) and a decrease in moss/lichen cover [75]. Our results, although resulting in some short term habitat changes and positive changes in lark abundance, could potentially be improved by altering the techniques used. For example, to obtain more lasting effects, it might be necessary to burn annually or every few years, a rate similar to that used by Native Americans. In addition, managers can attempt to achieve a hotter and more complete burn that is likely to result in the habitat conditions preferred by larks by burning at the end of the growing season when the vegetation has low moisture content, and by conducting burns when the relative humidity is also low [70]. It would also be helpful to understand whether higher intensity, but perhaps less frequent, or higher frequency, but less intense, fires produce habitat most suitable for larks. Focusing fire management on relatively nutrient poor soils or combining this technique with other methods such as appropriate post-fire seeding with suitable plants (appropriate structure and composition) or the selective use of herbicides could result in the sparse vegetation characteristics preferred by larks [70].

As we attempt to improve the quality of lark habitat, we recommend considering examining some of the nuances not addressed in this research. For example, larks select sites with low moss/lichen cover yet they have been observed eating moss sporophytes suggesting potential benefits of some moss species; non-native perennial forbs seem to have a positive influence on nest success yet we suspect that it is not necessarily important that the plant is not native, what is likely important is the interaction between plant architecture and bird behavior that results in lower predation rates.

## Conclusion

In this study, we moved beyond examining habitat use to assessing habitat selection by streaked horned larks. We accomplished this by examining habitat characteristics in both low and high lark density areas and by comparing nest sites with nearby random sites. This approach allowed us to recognize that larks are selective in their habitat use at both the nest site and territorial scales. At both scales, larks appear to be avoiding areas with dense moss and lichen. Interestingly, there are more differences than similarities in the variables selected at the nest and territorial scales (compare the nest and territory differences in Table 4). For example, areas that are sparsely vegetated by relative short vegetation are used preferentially within a given breeding site. In contrast, at a smaller nest-site scale females appear to be selecting patches within this landscape with more perennial forbs and greater vegetation density for their nests. Importantly, nest site selection has reproductive implications suggesting the importance of considering this factor in habitat restoration activities. Because these results are tentative and largely correlative, we recommend moving forward with management actions (e.g., creating forb rich patches within territories) in a research framework. Although the habitat and lark response relationships to prescribed fire were relatively weak, they were in the direction preferred by larks and therefore may be an area of future research and management. In our experience, conducting conservation actions in a research context provides valuable information for the species recovery process. Our results emphasize the importance of considering spatial scale and the potential reproductive consequences of habitat selection in habitat studies, especially because there is no guarantee that the presence of individuals in a given habitat is positively related to habitat quality [76–78].

## Supporting Information

S1 Table AICc table for choosing baseline model. The best supported model included nest age (quadratic) only.
(DOCX)
