# Supplementary material for: Considering Spatial Scale and Reproductive Consequences of Habitat Selection when Managing Grasslands for a Threatened Species
Source: PLoS One. 2016 Jun 20;11(6):e0156330. doi: 10.1371/journal.pone.0156330 (PMC4913950; doi:10.1371/journal.pone.0156330)
Supplement: S1 Table — The best supported model included nest age (quadratic) only. (DOCX) [file pone.0156330.s001.docx]

**S1 Table**. **AICc table for choosing baseline model.** The best supported model included nest age (quadratic) only.

| **Model** | **Conv** | **n** | **k** | **n-ess** | **AIC** | **AICc** | **DeltaAICc** | **Weight** | **ModLik** |
| --- | --- | --- | --- | --- | --- | --- | --- | --- | --- |
| Age (Quadratic) | Yes | 619 | 3 | 2269 | 699.469 | 699.480 | 0.000 | 0.320 | 1.000 |
| Age (Quadratic)  + Habitat (2 categories) | Yes | 619 | 4 | 2269 | 700.700 | 700.718 | 1.238 | 0.172 | 0.538 |
| Age (Quadratic)  + Habitat (4 Categories) | Yes | 619 | 6 | 2269 | 700.932 | 700.969 | 1.489 | 0.152 | 0.475 |
| Age (Quadratic)  + Year (Fixed) | Yes | 619 | 6 | 2269 | 703.815 | 703.852 | 4.372 | 0.036 | 0.112 |
| Age (Linear) | Yes | 619 | 3 | 2269 | 713.266 | 713.276 | 13.796 | 0.000 | 0.001 |
| intercept only | Yes | 619 | 1 | 2269 | 717.990 | 717.992 | 18.512 | 0.000 | 0.000 |
| Age (Linear) | Yes | 619 | 2 | 2269 | 718.037 | 718.042 | 18.562 | 0.000 | 0.000 |
| Date (linear) | Yes | 619 | 2 | 2269 | 718.167 | 718.172 | 18.692 | 0.000 | 0.000 |
| Habitat Types (2 categories) | Yes | 619 | 2 | 2269 | 719.524 | 719.529 | 20.049 | 0.000 | 0.000 |
| Habitat Types (4 categories) | Yes | 619 | 4 | 2269 | 719.526 | 719.543 | 20.064 | 0.000 | 0.000 |
| Year (fixed) | Yes | 619 | 5 | 2269 | 719.524 | 719.550 | 20.070 | 0.000 | 0.000 |
| Site-Year (Random) | Yes | 619 | 2 | 2269 | 719.933 | 719.939 | 20.459 | 0.000 | 0.000 |
| Year (random) | Yes | 619 | 2 | 2269 | 719.990 | 719.995 | 20.515 | 0.000 | 0.000 |
| Site (random) | Yes | 619 | 2 | 2269 | 719.990 | 719.995 | 20.515 | 0.000 | 0.000 |
| Date (Quadratic) | Yes | 619 | 3 | 2269 | 720.157 | 720.168 | 20.688 | 0.000 | 0.000 |
| Site (fixed) | Yes | 619 | 7 | 2269 | 724.819 | 724.869 | 25.389 | 0.000 | 0.000 |
